# Thyroid autoimmunity and hypothyroidism are associated with deep molecular response in patients with chronic myeloid leukemia on tyrosine kinase inhibitors

**DOI:** 10.1007/s40618-021-01613-5

**Published:** 2021-07-20

**Authors:** R. Rodia, F. Pani, G. Caocci, G. La Nasa, M. P. Simula, O. Mulas, F. Velluzzi, A. Loviselli, S. Mariotti, F. Boi

**Affiliations:** 1 Endocrinology Unit, Department of Medical Sciences and Public Health, University of Cagliari, Azienda Ospedaliero-Universitaria di Cagliari, SS 554, Bivio per Sestu, Monserrato, 09042 Cagliari, Italy; 2 Division of Immunology, Department of Pathology, The Johns Hopkins School of Medicine, Baltimore, MD USA; 3 Ematology and CTMO, Businco Hospital, Azienda Ospedaliera Brotzu, Cagliari, Italy

**Keywords:** Tyrosine kinase inhibitors, Thyroid abnormalities, Chronic myeloid leukemia, Thyroid autoimmunity, Molecular response

## Abstract

**Purpose:**

Thyroid alterations including de novo appearance of thyroid autoimmunity are adverse effects of tyrosine kinase inhibitors, used in solid and hematologic cancer therapy, but the relationship between thyroid alterations during this treatment and the outcome of chronic myeloid leukemia remains unclear. Aim of this study was to investigate whether the presence of thyroid alterations may affect the clinical outcome of chronic myeloid leukemia on tyrosine kinase inhibitors.

**Methods:**

We evaluated thyroid function and autoimmunity in 69 chronic myeloid leukemia patients on long-term therapy looking at the association between thyroid abnormalities and disease molecular response.

**Results:**

Overall, 24 of 69 (34.8%) had one or more thyroid abnormalities during therapy. A high percentage of patients (21/69, 30.4%) showed thyroid autoimmunity (positive thyroid autoantibodies with ultrasound hypoechogenicity), while clinical and subclinical hypothyroidism and subclinical hyperthyroidism were, respectively, found in 4 of 69 (5.8%) and 3 of 69 (4.3%) of cases. Second-generation tyrosine kinase inhibitors resulted significantly associated (14/32, 43.7%) with Hashimoto’s thyroiditis, compared to first generation (7/37, 18.9%; *p* = 0.03). Interestingly, we also found a significant association between euthyroid (14/26, 53.8%) and hypothyroid Hashimoto’s thyroiditis (4/26, 15.4%) in patients with deep molecular response, as compared to euthyroid (3/43, 7%; *p* = 0.0001) and hypothyroid (0/43, 0%; *p* = 0.02) Hashimoto’s thyroiditis patients with major molecular response.

**Conclusions:**

Our study confirms and extends our knowledge on the tyrosine kinase inhibitors effects on thyroid, showing that thyroid autoimmunity is frequently observed in chronic myeloid leukemia patients on long-term therapy and is associated with a better oncological response.

## Introduction

Tyrosine kinase inhibitors (TKIs) block the phosphorylation pathways of cellular signaling proteins, essential for tumor cell proliferation [1]. Several endocrine side effects [2, 3] have been described with the increased use of TKIs in cancer therapy and thyroid alterations, mostly hypothyroidism, represents a well-known phenomenon [4–8]. TKI-induced thyroid alterations are caused by several direct mechanisms, such as thyroid damage ranging from mild follicular cells toxicity [9] to destructive thyroiditis [10–12], inhibition of thyroid peroxidase, blocking iodine uptake, and increased thyroid hormone clearance [13–15]. TKIs may also induce indirect thyroid damage via their antiangiogenic activity [13, 16]. More recently, de novo appearance of serum thyroid autoantibodies has been observed in almost one-third of oncologic patients followed before and during sunitinib therapy, suggesting that triggering of thyroid autoimmunity may be involved in TKIs-induced thyroid dysfunction [17]. Both hypothyroidism and thyroid autoimmunity induced by TKIs in patients with different solid tumors have been found to be associated with a better oncologic response, although the underlying mechanism(s) remains to be elucidated [17, 18].

TKIs are routinely employed in the treatment of Philadelphia chromosome-positive chronic myeloid leukemia (Ph-positive CML). These TKIs specifically target the oncogenic activity of the breakpoint cluster region (BCR)-Abelson murine leukemia viral oncogene homolog (ABL) protein in patients with CML. Four TKIs have been approved for first-line therapy (FLT) CML: imatinib (first-generation TKI), dasatinib, nilotinib, and bosutinib (second-generation TKI). In cases of failure/resistance, all second-generation TKIs are effective, but the criteria for the choice of the second-line therapy (SLT) are patient related and depend on age, comorbidities, and toxicity of FLT with TKIs [19]. Besides a recent review of cases reported by the U.S. Food and Drug Administration [20], only one retrospective monocentric study has been carried out on thyroid function in patients with CML on TKIs treatment [8], showing that thyroid dysfunction (mainly subclinical) is common during both first-generation and second-generation TKIs therapy. However, no data are available on the relevance of thyroid autoimmunity in TKIs-induced thyroid dysfunction in patients with CML and on the potential relationship between thyroid dysfunction/autoimmunity and the response to treatment.

With this concept in mind, we assessed thyroid function and autoimmunity in relation to the outcome of disease in a series of CML patients during treatment with first- and second-generation TKIs.

## Materials and methods

### Patients

This study enrolled an original cohort of 77 adult patients with Ph-positive CML in chronic phase treated with imatinib, nilotinib, or dasatinib as FLT or SLT.

**Fig. 1:**
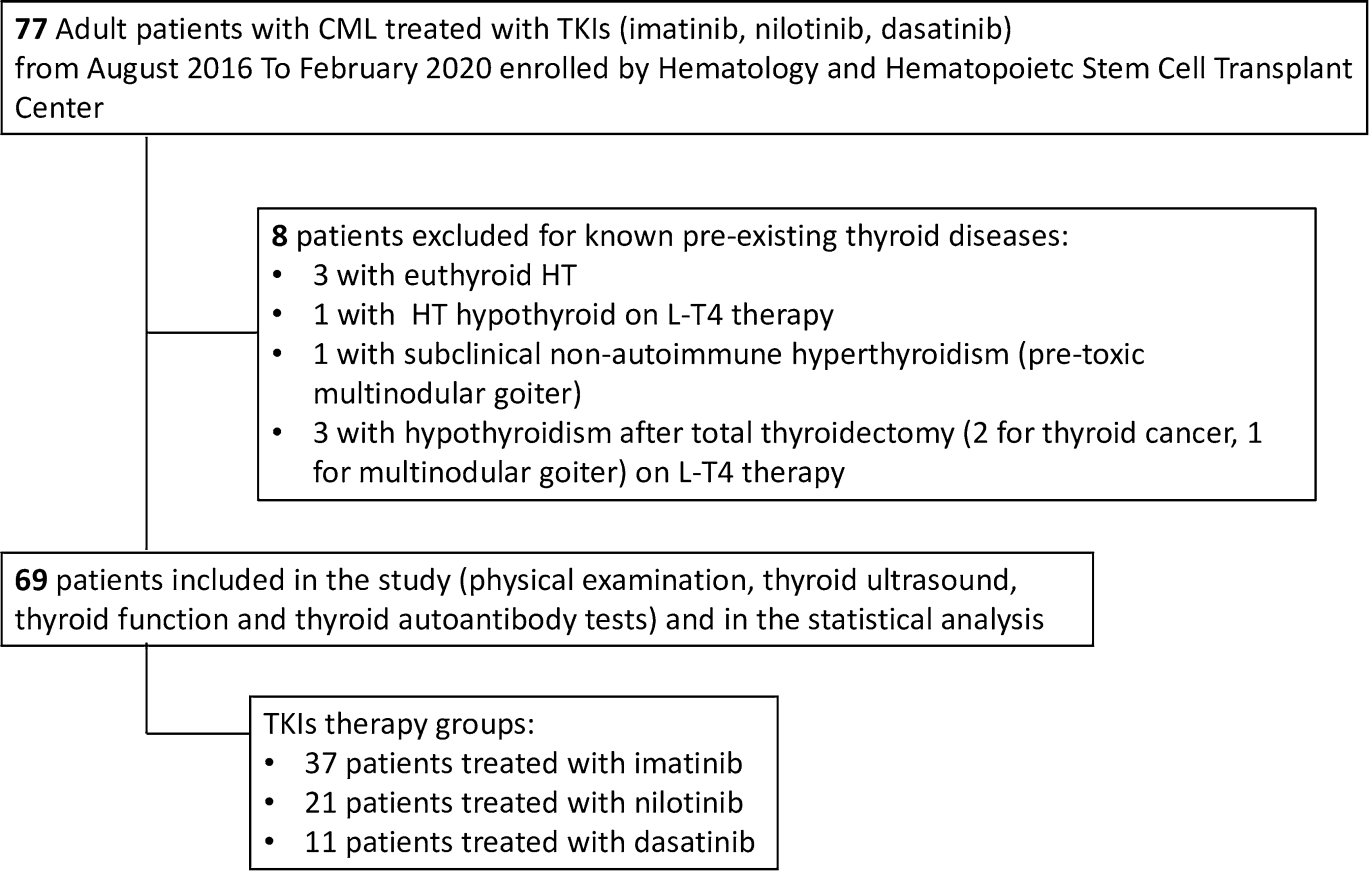
Flow diagram showing protocol followed in patients’ recruitment

Written informed consent was obtained from each patient after full explanation of the purpose and nature of all procedures used. The Institutional Review Board of University Hospital of Cagliari reviewed and approved the protocol of this study. Patients were treated with imatinib 400 mg once a day, nilotinib 300 mg two times a day, and/or dasatinib 100 mg once a day, and dose reductions were performed in case of toxicity. Determination of disease stage and response criteria were established according to standard criteria [21]. Although a baseline thyroid function and autoimmunity were not systematically performed before starting TKIs therapy, all patients were screened by a careful evaluation of familiar history of thyroid diseases, treatment with thyroid drugs, and previous thyroid records (thyroid hormones and/or auto-antibodies), independently made before treatment. Between August 2016 and February 2020, clinical records of these CML patients were collected and refined by the exclusion of patients with preexisting thyroid alterations or cases treated with thyroid medications that might interfere with thyroid status. As detailed in Fig. 1, we excluded from further analysis 8 cases: 3 patients with euthyroid HT, 1 with hypothyroid HT, 1 with pre-toxic adenoma and 3 with post-surgical hypothyroidism (2 for thyroid cancer and 1 for multinodular goiter). The timing of thyroid parameters measurements during TKIs therapy was represented by a median of 48 months (range 3–216). Since this cross-sectional study consisted in a single thyroid data collection in a definite time, no evaluation of differences in the time of thyroid alterations appearance, among different TKIs, was performed.

### Treatment response of CML

The evaluation of CML treatment response, according to the standards established by the European LeukemiaNet, is based on hematological (blood count and spleen volume), cytogenetic (presence of metaphases Ph + in marrow cells), and molecular (evidence of BCR-ABL transcript) responses [21]. The progressive responses, achieved on TKI treatment, are the following: complete hematological, complete cytogenetic, major molecular response (MMR), and deep molecular response (DMR), these latter corresponding to progressive lower levels of transcript. Molecular responses (MR) represent the most sensitive method to assess the state of the disease and in particular to measure residual disease. MR assessment is performed by quantitative RT-PCR of BCR-ABL transcript on leukocytes isolated from a peripheral blood sample. It indicates the variation of this transcript, periodically evaluated during TKI therapy, in comparison to the value detected at the time of the diagnosis. BCR-ABL transcript levels should be expressed according to the international scale (BCR-ABL1 IS%) to guarantee the comparability of results among different laboratories.

Since MR represents the most effective method in the evaluation of CML treatment response, thyroid alterations were correlated with MMR (BCR-ABL transcript levels ≤ 0.1% in IS) and DMR (BCR-ABL transcript levels ≤ 0.01% in IS) states.

### Thyroid function assays

Hormonal and thyroid autoantibody assays were carried out using commercial kits. Thyroid function tests included serum thyroid-stimulating hormone (TSH), free triiodothyronine (fT3), free thyroxine (fT4) (normal values: TSH 0.55–4.78 µUI/ml; fT3 2.3–4.2 pg/ml; fT4 0.89–1.76 ng/dl), anti-thyroid autoantibodies (ATA) against thyroid peroxidase (TPOAb) and thyroglobulin (TgAb) (both positive when > 60 UI/ml). All these assays were performed by ultrasensitive automatic chemiluminescent method (ADVIA Centaur CP Immunoassay System, Siemens Healthineers, Erlangen, Germany). TSH-receptor antibodies (TRAb, normal values < 1.86 IU/l) were assayed in hyperthyroid patients by an automated chemiluminescent method (Elecsys® Anti-TSHR, Roche Diagnostics Ltd, Rotkreuz, Switzerland). All the assays were performed in the laboratory of “Duilio Casula” University Hospital of Cagliari.

### Thyroid ultrasound

Thyroid ultrasound (US) was performed using a Siemens Antares color Doppler system equipment (Siemens, Medical Solutions, Issaquah, WA). All patients were evaluated by US examination. According to thyroid ultrasound criteria [22–24], parenchymal echogenicity was classified in normoechoic or hypoechoic pattern (low, moderate, and marked, these latter being a typical US feature of thyroid autoimmunity). Based on suspicious ultrasound features, thyroid nodules found during the study were submitted to fine needle aspiration cytology (FNAC) [24, 25].

### Definition of hyperthyroidism, hypothyroidism, and autoimmune thyroid disease

Hyperthyroidism and hypothyroidism were diagnosed according to the current guidelines [26, 27]. Overt hyperthyroidism was defined by high serum fT4 or fT3 and undetectable serum TSH concentration, while overt hypothyroidism as low serum fT4 with elevated serum TSH concentration. Subclinical hyperthyroidism was defined as serum TSH below the lower limit of normal with fT4 and fT3 concentration in their reference ranges, while subclinical hypothyroidism was diagnosed by serum TSH above the upper limit of normal with fT4 concentration within its reference range. The diagnosis of autoimmune thyroid disease (Hashimoto’s thyroiditis [HT] or Graves’ disease [GD]) was based on the presence of typical US features (hypoechoic pattern for both HT and GD, increased diffuse vascularity for GD), serum positive TgAb and TPOAb (for HT), TRAb (for GD), and increased serum TSH (for HT).

### Statistical analysis

All statistical analyses were performed using Graphpad® Software Inc. (San Diego, USA). Categorical variables were summarized as the counts and percentages (%). Differences between the groups were analyzed by Pearson’s chi-square test and the Fisher’s exact test. Statistical significance was defined as *p* values < 0.05.

## Results

### Patient characteristics

As summarized in Fig. 1, after the exclusion of 8 patients with preexisting thyroid alterations or treated with thyroid medications, the remaining 69 cases (44 [63.7%] males and 25 [36.3%] females) were considered for the present study. At the time of the diagnosis, the median age of all patients was 52 years (range 18–78). On the basis of the drug employed in the CML treatment, patients were subdivided into three groups (Table 1). Thirty-seven (53.6%) received imatinib (first-generation TKI), 21 (30.4%) received nilotinib, and 11 (16%) dasatinib (both belonging to second-generation TKIs). Forty-nine (71%) patients were on FLT with TKIs (36/49 on imatinib and 13/49 on second-generation drugs). Due to primary failure, 20/69 (29%) patients were on SLT (1/20 on imatinib and 19/20 on second-generation TKIs); for further details of previous treatment, see Table 1. Median therapy duration of CML was 48 months (range 3–216). As shown in Table 1, 43 (62.3%) patients showed MR and 26 (37.7%) cases showed DMR and, as expected by the literature [28–30], patients on nilotinib and dasatinib showed more frequently DMR (17/32, 53.1%) than those on imatinib (9/37, 24.3%, *p* = 0.02).

**Table 1:** CML patients’ characteristics and thyroid alterations observed during TKIs therapy

Patient characteristics		Total	Imatinib	Nilotinib	Dasatinib
*n*		69	37	21	11
Male (%)		44 (63,7)	23 (62.2)	16 (76.2)	5 (45.5)
Female (%)		25 (36.3)	14 (37.8)	5 (23.8)	6 (54.5)
Median age at diagnosis (range)		52 (18–78)	55 (24–78)	46 (18–70)	52 (26–67)
First line therapy (%)		49 (71)	36 (97.3)	8 (38)	5 (45.4)
Second line therapy (%)		20 (29)	1 (2.7)	13 (62)	6 (54.6)
	Imatinib (%)	17 (24.6)	NA	11 (52.4)	6 (54.6)
Previous treatment:	Dasatinib (%)	1 (1.4)	0	1 (4.7)	NA
	Interferon (%)	2 (2.9)	1 (2.7)	1 (4.7)	0
Median therapy duration (months, range)		48 (3–216)	48 (3–216)	48 (3–156)	72 (3–216)
Normal thyroid function (%)		45 (65.2)	28 (75.8)	11 (52.4)	6 (54.5)
Total thyroid alterations (%)		24 (34.8)	9 (24.3)	10 (47.6)	5 (45.5)
HT− (%)		48 (69.6)	30 (81)	12 (57.1)	6 (54.5)
HT+ (%)		21 (30.4)	7 (18.9)	9 (42.8)	5 (45.5)
ATA+ (%)		21(30.4)	7 (18.9)	9 (42.8)	5 (45.5)
Moderate US hypoecogenicity (%)		10 (14.5)	5 (13.5)	3 (14.3)	2 (18.2)
Marked US hypoecogenicity (%)		11 (15.9)	2 (5.4)	6 (28.6)	3 (27.3)
Hypothyroidism (%)^β^		4 (5.8)	1 (2,7)	2 (9.5)	1 (9)
Subclinical (%)		2 (2.9)	1 (2.7) ^δ^	1 (4.7) ^γ^	0
Overt (%)		2 (2.9)	0	1 (4.7) ^δ^	1 (9) ^δ^
Subclinical Hyperthyroidism (%)^℧^		3 (4.3)	2 (5.4)	1 (4.7)	0
MMR (%)		43 (62.3)	28 (75.7)	10 (47.6)	5 (45.4)
DMR (%)		26 (37.7)	9 (24.3)	11 (52.4)	6 (54.6)

*CML* Chronic myeloid leukemia, *TKIs* Tyrosine kinase inhibitors, *NA* not applicable, *MMR* major molecular response, *DMR* deep molecular response, *HT* Hashimoto’s thyroiditis, *ATA* anti-thyroid antibodies, − absence, + presence, β all ATA+, *Ʊ* all ATA−, γ with moderate ultrasound (US) hypoechogenicity, δ with marked US hypoechogenicity

### Thyroid alterations during TKIs therapy

Twenty-four (34.8%) patients showed one or more thyroid abnormalities on TKIs therapy. As shown in Table 1, thyroid alterations were found in 9/37 (24.3%) patients treated with imatinib, and in a higher proportion of those treated with nilotinib (10 of 21; 47.6%) and dasatinib (5 of 11; 45.5%). These thyroid abnormalities (autoimmunity, function, and nodules) are detailed below.

#### Thyroid autoimmunity

Twenty-one patients (30.4%) resulted TPOAb and/or TgAb positive (ATA +), of whom 10 (47.6%) showed moderate US hypoechogenicity and 1 had subclinical hypothyroidism. The remaining 11 (52.4%) ATA + cases had marked US hypoechogenicity and 1 of them had subclinical and 2 overt hypothyroidism. Thus, all the above 21 ATA + patients fulfilled the diagnostic criteria of HT. The prevalence of HT was 7 out of 37 patients (18.9%) in the imatinib group, 9 out of 21 (42.8%) in the nilotinib group, and 5 out of 11 (45%) in the dasatinib group, see Table 1. HT prevalence resulted significantly higher in patients taking second-generation TKIs (nilotinib and dasatinib) than in those on imatinib (43.75% vs 18.9%; *p* = 0.03). To exclude the potential impact of previous treatment on thyroid autoimmunity in patients on SLT, we compared the prevalence of HT between SLT patients *vs* those on FLT. No statistical difference in HT prevalence was observed between FLT patients on second-generation TKIs compared to those in SLT (38.5% *vs* 47.4%; *p* = 0.7), suggesting that previous treatment did not increase HT prevalence. Since imatinib was almost exclusively (97.3%) administered in FLT, no comparison was performed *vs* SLT patients.

Finally, the remaining 48 patients (69.6%) did not show any features of HT (HT-), resulting TPOAb and/or TgAb negative with normal echogenicity at US.

#### Thyroid function

As displayed in Table 1, hypothyroidism was detected in 4 (5.8%) patients. In particular, overt hypothyroidism was observed in 2 (2.9%) patients that underwent l-thyroxine replacement therapy and subclinical hypothyroidism was detected in the remaining 2 (2.9%) cases, all belonging to HT group (4/21, 19%). Three/4 hypothyroid patients were on second-generation TKIs (2 overt hypothyroidism, 1 on Nilotinib, and 1 on Dasatinib; 1 subclinical hypothyroidism on Nilotinib), while only one (subclinical hypothyroidism) was on Imatinib. Subclinical non-autoimmune hyperthyroidism was found in 3 (4.3%) patients, while no overt hyperthyroidism was observed. Two/3 subclinically hyperthyroid patients were on first-generation TKI (imatinib) and a diagnosis of pre-toxic multinodular goiter was made by thyroid US and scintigraphy. The remaining patient, treated with nilotinib, showed at US reduced thyroid volume, mild hypoechogenicity and reduced vascularization; absent uptake was observed at thyroid scintiscan, leading to the presumptive diagnosis of TKI-induced destructive thyroiditis.

### Association between thyroid alterations and molecular response in CML patients

When patients were subdivided on the basis of presence/absence of thyroid alterations, we found higher DMR (19/26, 73.1%) and lower MMR (5/43; 11.6%) in thyroid alterations group than DMR (7/26, 26.9%) and MMR (38/43; 88.4%; *p* = 0.0001) observed in patients without thyroid alterations. As shown in Table 2, the single thyroid alteration significantly associated with DMR than MMR was HT (69.2% vs 7%; *p* = 0.0001), irrespective whether euthyroid (53.8% vs 7%; *p* = 0.0001) or hypothyroid (15.4% vs 0%; *p* = 0.02).

**Table 2:** Correlation between thyroid alterations and degree of CML molecular response during TKIs therapy

Thyroid alterations	DMR	MMR	*p*
Total			
No patients (%) positive	19/26 (73.1)*	5/43 (11.6)	*0.0001 vs MMR
No patients (%) negative	7/26 (26.9)	38/43 (88.4)	
HT			
No patients (%) positive	18/26 (69.2)*	3/43 (7)	*0.0001 vs MMR
No patients (%) negative	8/26 (30.8)	40/43 (93)	
Euthyroid HT			
No patients (%) positive	14/26 (53.8)*	3/43 (7)	*0.0001 vs MMR
No patients (%) negative	12/26 (46.2)	40/43 (93)	
Hypothyroid HT			
No patients (%) positive	4/26 (15.4)*	0/43 (0)	*0.02 vs MMR
No patients (%) negative	22/26 (84.6)	43/43 (100)	

*CML* Chronic myeloid leukemia, *TKIs* Tyrosine kinase inhibitors, *MMR* major molecular response, *DMR* deep molecular response, *HT* Hashimoto’s thyroiditis

**Fig. 2:**
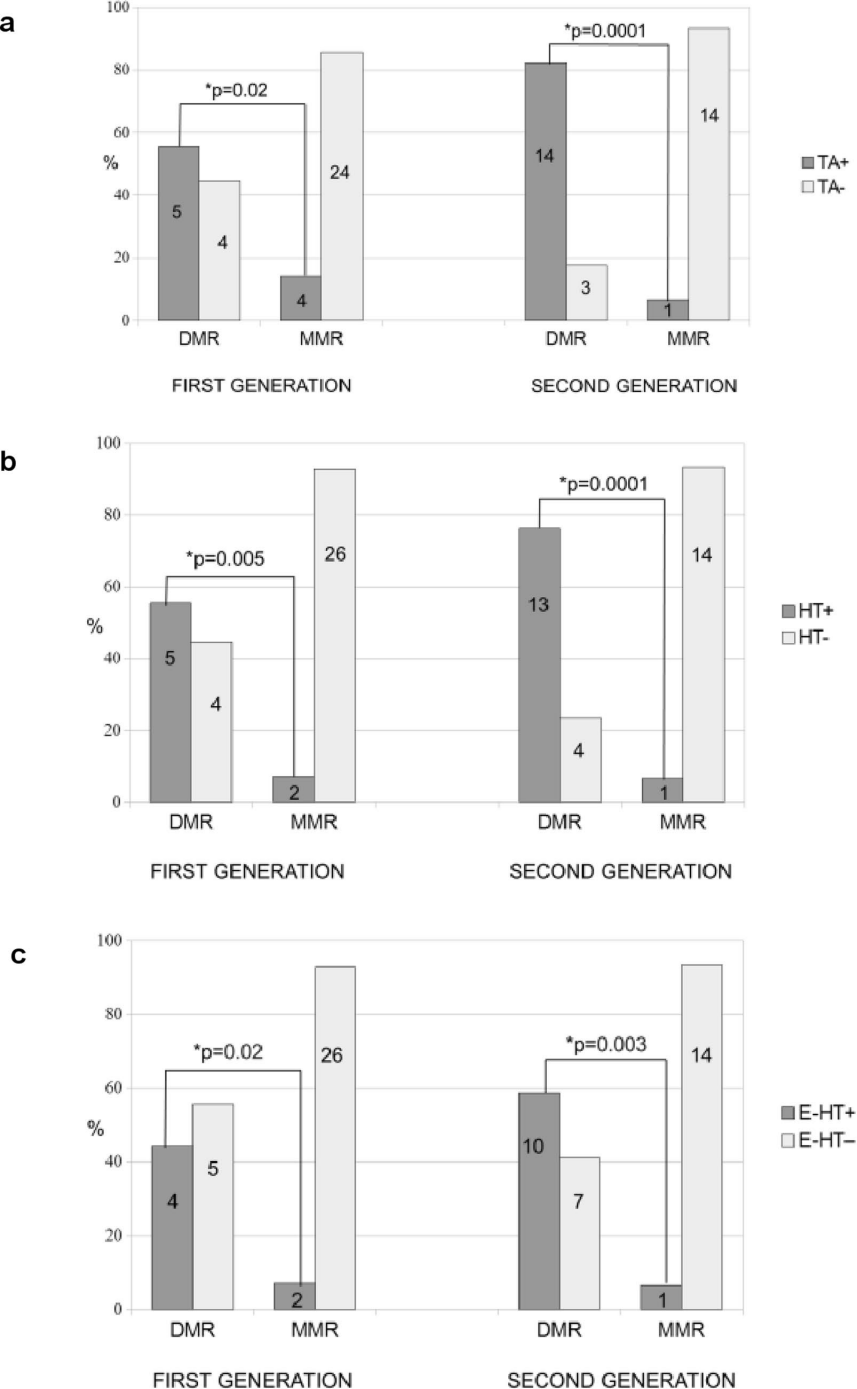
**a** Prevalence of thyroid alterations (TA+ presence; TA− absence) in deep molecular response (DMR) and in major molecular response (MMR) of chronic myeloid leukemia (CML), observed in patients treated with first- and second-generation tyrosine kinase inhibitors (TKIs). **b** Prevalence of Hashimoto’s thyroiditis (HT+ presence; HT– absence) in DMR and in MMR of CML, observed in patients treated with first- and second-generation TKIs. **c** Prevalence of euthyroid Hashimoto’s thyroiditis (E-HT+ presence; E-HT– absence) in DMR and in MMR of CML, observed in patients treated with first- and second-generation TKIs. The number of patients with thyroid abnormalities (TA, HT, and E-HT) is reported in each column of the figure

Furthermore, as shown in Fig. 2a, the higher prevalence of DMR vs MMR observed in thyroid alterations group was also maintained when first- generation (55.5% vs 14.3%; *p* = 0.02) and second-generation (82.3% vs 6.6%; *p* = 0.0001) TKIs, were separately evaluated. Similar results were also obtained for all HT patients on first- (55.5% vs 7.1%; *p* = 0.005) and second-generation (76.4% vs 6.6%; *p* = 0.0001) TKIs, see Fig. 2b, as well as for euthyroid HT on first- (44.4% vs 7.1%; *p* = 0.02) and second-generation (58.8% vs 6.6%; *p* = 0.003) TKIs, see Fig. 2c.

Finally, no association with DMR was found in the 3 patients with subclinical non-autoimmune hyperthyroidism and in the 24 patients with nodules at thyroid US (data not shown). Of these, 8 (33.3%) were selected on the basis of standard criteria [31] for FNAC, which was indicative of benign lesion (Thy 2) in 6 and of indeterminate follicular lesion (Thy 3) in the remaining 2 patients.

## Discussion

After the introduction of TKI therapy, hypothyroidism induced by imatinib has been frequently reported in patients with solid tumors [32–34], but only one monocentric study is available on the thyroid effects observed in CML patients treated with imatinib, nilotinib, and dasatinib [8]. This study showed a high prevalence of thyroid dysfunction (mainly mild subclinical hypothyroidism and transient destructive thyrotoxicosis, generally not requiring specific therapy). The most consistent mechanism of imatinib interference with thyroid function is an increase of thyroid hormone needs, by a rise of nondeiodination clearance through the induction of uridine diphosphate–glucuronosyltransferases. This is responsible of “consumption hypothyroidism”, mostly if not exclusively observed in thyroidectomized patients assuming l-thyroxine therapy [15]. The other effects (primary hypothyroidism and destructive thyrotoxicosis) observed in patients with thyroid in situ are due to several direct effects of TKIs on thyroid follicular cells [8, 35]. At difference with imatinib, the mechanisms involved in thyroid side effects of second-generation TKIs (nilotinib and dasatinib) have not been so far investigated.

The present cross-sectional study analyzed thyroid function, thyroid autoimmunity, and thyroid ultrasound findings in all patients with CML, during TKI treatment. The results showed a very high rate (34.8%) of thyroid alterations during TKIs treatment and this prevalence was higher for nilotinib and dasatinib (47.6% and 45.5%, respectively) compared to Imatinib (24.3%). This finding is in keeping with the study by Kim et al. [8], where thyroid dysfunction was very frequently observed in CML patients on TKIs therapy with a prevalence of 25%, 55% and 70% with imatinib, nilotinib, and dasatinib, respectively. In Kim et al.’s study, autoimmune thyroiditis (as assessed by positive ATA) was only 7%, while in our series, the prevalence of euthyroid or hypothyroid Hashimoto’s thyroiditis (as assessed by immunological, functional, and US criteria) was much higher (30.4%). The difference could be due to the inclusion of systematic US, thyroid function and ATA measurements in our protocol, while in the Kim’s paper [8], ATA were assayed only in patients with increased serum TSH. It is evident that only systematical use of US, thyroid function, and ATA assays allows the detection of euthyroid HT. Actually, the prevalence of HT was very high in our series, especially in patients treated with second-generation TKIs (nilotinib and dasatinib) as compared to imatinib. Furthermore, this result was also confirmed by the comparison between FLT vs SLT patients on second-generation TKIs, which suggested that previous treatment did not affect HT prevalence. However, an important limitation of the present study is represented by the lack of baseline thyroid function and autoimmunity data before starting TKIs therapy. However, the prevalence of HT in our cohort of TKIs-treated CML patients is higher than that expected in the general population [36–38], even considering the high frequency of autoimmune diseases in Sardinia [17, 38–42]. Moreover, de novo appearance of serum ATA has been previously documented in cancer patients on TKIs therapy, at least with sunitinib. Thus, it is conceivable that the high rate of HT found in our cohort of CML patients might be the consequence of chronic TKIs treatment, especially for nilotinib and dasatinib.

Focusing on thyroid dysfunction, the prevalence of hypothyroidism found in our research (5.8%) was similar to that found by Kim et al. (8%). However, all our hypothyroid cases were associated with HT, while this association in Kim's study was lower (22%). Another difference from Kim’s study was the fact that we did not find any case of transient non-autoimmune hypothyroidism, while a significant percentage (16%) of this condition was reported by Kim et al. This difference could be explained by the nature of our cross-sectional study, which collected data of CML patients only in a definite time during TKI treatment, while Kim’s study retrospectively evaluated multiple assessments of thyroid function at different times during the follow-up.

Although the prevalence of hyperthyroidism in our research (4.3%) is lower than that described by Kim et al. (29%), it should be noted that in the latter study, most (23%) cases were represented by transient hyperthyroidism. In contrast, we only found a single case of a destructive thyroiditis induced by nilotinib, in keeping to a recent report [35], while the remaining 2 patients treated with imatinib showed a pre-toxic multinodular goiter that may have been present before therapy rather than being related to TKI; no cases of autoimmune hyperthyroidism were observed. This discrepancy could be, at least in part, attributed by the nature of our cross-sectional study, that might underestimate the cases of transient hyperthyroidism and to the high prevalence of functional thyroid autonomy (pre-toxic multinodular goiter, pre-toxic adenoma) in iodine-deficient areas, such as Sardinia [36, 43].

The potential effect of TKIs-related thyroid abnormalities on progression-free survival (PFS) and overall survival (OS) of solid carcinoma has been recently reported in some observational studies, in particular by Lechner et al. [18]. They demonstrated an improvement in OS among TKI-treated patients (including imatinib, nilotinib, and dasatinib) who developed overt hypothyroidism [18]. Furthermore, an association between new appearance of ATA positivity and longer PFS has also been described in TKI-treated patients with solid carcinomas [17, 44].

At difference with solid cancers, to our knowledge, no study has been carried out so far to evaluate the effects of thyroid abnormalities on the clinical course of CML patients on TKIs treatment. The results obtained in the present investigation provide clear evidence of a higher prevalence of HT and hypothyroidism in patients with better outcome (DMR). Of particular relevance is the finding that a higher remission rate was observed with both first- and second-generation TKIs and that thyroid autoimmunity, rather than dysfunction, was critically associated with better MR. Indeed, the association of DMR and HT remained highly significant when the analysis was limited to euthyroid patients and no association was found with any degree of hyperthyroidism and/or the presence of thyroid nodules. Whether and to what extent the progression from euthyroid to hypothyroid HT might further mark the responsiveness of CML patients to TKIs, as shown in solid tumors [17, 18, 44], remains matter of speculation.

The high prevalence of thyroid autoimmunity found in patients with a better outcome of CML may support the hypothesis that a widespread activation of the immune system may predispose to HT development and, at the same time, may contribute to the control of tumor growth by a systemic immune response to cancer [17, 44]. In keeping to this hypothesis is the study of Steegmann et al. [45], that demonstrated a potential immuno-activating effect of dasatinib inducing a major molecular response in CML patients. The early detection of thyroid autoimmunity and autoimmune hypothyroidism, not only could be useful to identify patients who developed HT, but might be also considered an important biomarker of TKI therapeutic efficacy.

In conclusion, our study confirms and extends our knowledge on TKIs-related thyroid alterations, showing that thyroid dysfunction and autoimmunity are frequently observed in CML patients on TKIs therapy and may predict a better oncological response. These thyroid alterations are generally subclinical and do not require dose reduction or discontinuation of TKIs. Further larger multicentric prospective controlled studies are needed to confirm the clinical relevance of our research.

## Data Availability

All data generated or analyzed during this study are included in this published article.
